# Overexpression of the protein phosphatase 2A regulatory subunit a gene *ZmPP2AA1* improves low phosphate tolerance by remodeling the root system architecture of maize

**DOI:** 10.1371/journal.pone.0176538

**Published:** 2017-04-27

**Authors:** Jiemin Wang, Laming Pei, Zhe Jin, Kewei Zhang, Juren Zhang

**Affiliations:** 1 School of Life Sciences, Shandong University, Ministry of Education Key Laboratory of Plant Cell Engineering and Germplasm Enhancement, Jinan, China; 2 Department of Biotechnology, School of Biological Science and Technology, University of Jinan, Jinan, China; Henan Agricultural University, CHINA

## Abstract

Phosphate (Pi) limitation is a constraint for plant growth and development in many natural and agricultural ecosystems. In this study, a gene encoding *Zea mays* L. protein phosphatase 2A regulatory subunit A, designated *ZmPP2AA1*, was induced in roots by low Pi availability. The function of the *ZmPP2AA1* gene in maize was analyzed using overexpression and RNA interference. *ZmPP2AA1* modulated root gravitropism, negatively regulated primary root (PR) growth, and stimulated the development of lateral roots (LRs). A detailed characterization of the root system architecture (RSA) in response to different Pi concentrations with or without indole-3-acetic acid and 1-N-naphthylphthalamic acid revealed that auxin was involved in the RSA response to low Pi availability. Overexpression of *ZmPP2AA1* enhanced tolerance to Pi starvation in transgenic maize in hydroponic and soil pot experiments. An increased dry weight (DW), root-to-shoot ratio, and total P content and concentration, along with a delayed and reduced accumulation of anthocyanin in overexpressing transgenic maize plants coincided with their highly branched root system and increased Pi uptake capability under low Pi conditions. Inflorescence development of the *ZmPP2AA1* overexpressing line was less affected by low Pi stress, resulting in higher grain yield per plant under Pi deprivation. These data reveal the biological function of *ZmPP2AA1*, provide insights into a linkage between auxin and low Pi responses, and drive new strategies for the efficient utilization of Pi by maize.

## Introduction

Phosphorus (P) is an essential macronutrient for plants. It serves both as a component of cellular materials, such as nucleic acids, membranes, and ATP, and as a key component in the regulation of numerous enzymatic activities and metabolic processes [1]. Despite its abundance in natural soil, inorganic phosphate (Pi) is often limiting for plants due to its strong affinity for cations and its rapid conversion to organic forms that are not readily available for plant uptake. To prevent P starvation, modification of the morphology of the RSA is one adaptive mechanism to increase exploration activity to facilitate plant acquisition of more Pi from the soil [2–5]. In *Arabidopsis*, low Pi availability attenuates PR growth, facilitates LR initiation and development, and increases root hair generation [6, 7]. The response of the RSA to Pi availability can be distinct between the dicotyledon *Arabidopsis* and monocotyledon *Zea mays* because the RSA and cellular organization patterning of individual roots can differ significantly between the two species [8–10]. Variation also exists among *Zea mays* genotypes in terms of the effects of Pi starvation on the RSA [11–14].

The phytohormone auxin is believed to play a central role in root architecture modifications in response to Pi deprivation [2, 15–20]. The application of exogenous auxin inhibits PRs and promotes LRs in *Arabidopsis*, mimicking the alteration of the RSA induced by Pi deficiency [2, 21]. Nacry et al. [18] proposed that the effects of Pi on the RSA, such as the shortening of PRs, inhibition of LR primordium initiation, and the stimulation of initiated LR primordium activation, could be caused by the redistribution of auxin through changes in auxin transport rather than auxin synthesis.

Polar auxin transport (PAT) is mediated by auxin influx and efflux carrier proteins; their polar cellular localization determines the directional transport of auxin between cells. In plants, PIN-FORMED (PIN) proteins are auxin efflux carriers [22–29]. The polar intracellular localization of these proteins is determined by reversible phosphorylation in the central hydrophilic loop through the reciprocal regulation of PID kinase and PP2A [30–32].

PP2A is a major Ser/Thr protein phosphatase consisting of multiple subunits, including the catalytic C subunit, the scaffolding A subunit, and the regulatory B subunit. The PP2A catalytic (C) subunit binds to the A subunit to form a constant dimeric core, which can be associated with different members of the variable B subunit family to produce several species of holoenzymes with distinct properties and functions [33, 34]. In *Arabidopsis*, three genes encode A subunits: *PP2AA1* encodes the A1 isoform (also known as *RCN1*), *PP2AA2* encodes the A2 isoform, and *PP2AA3* encodes the A3 isoform [35]. The biological functions of the AtPP2AA isoforms partially overlap, but RCN1 plays a cardinal role, while the functions of PP2AA2 and PP2AA3 are only unmasked when RCN1 is absent. Loss of *AtPP2AAs* function causes abnormities, such as root agravitropism, cotyledon defects and root meristem collapse throughout seedling development, as well as aberrations in embryo development. The rosette leaf growth, stem elongation, and reproductive development of adult phenotypes also require the functioning of the A subunits [32, 35–39].

The *rcn1* mutant was identified in a screening process for a root-curling response in the presence of NPA [40, 41]. Roots of *rcn1* seedlings exhibit elevated basipetal auxin transport and a significant delay in gravitropism [39, 42]. It is clear that the phenotypes observed in *rcn1* roots are caused by reduced PP2A activity because wild-type (WT) seedlings treated with low doses of protein phosphatase inhibitors can phenocopy *rcn1* [43]. To date, most advances in the study of PP2AAs have focused on the model plant *Arabidopsis*, and knowledge of these important protein phosphatases in monocot crops remains limited.

Pharmacological protein phosphatase inhibitor studies, the expression profiles of PP2A subunits under stress conditions, and the use of loss-of-function mutants or overexpression/silencing of specific PP2A subunit isoforms suggest that PP2As participate in biotic and abiotic stress signaling pathways [44]. Two PP2A catalytic subunit genes in rice (*Oryza sativa*) are differentially expressed in organs and respond to drought, salinity, and heat stresses [45]. Additionally, cold stress down-regulates the mRNA levels of *LePP2Ac1*, *LePP2Ac2*, and *LePP2Ac3* in tomato (*Solanum lycopersicum*) leaves. Salt stress induces the expression of *StPP2Ac1*, *StPP2Ac2a*, *StPP2Ac2b*, and *StPP2Ac3* in potato (*Solanum tuberosum*) leaves [46]. Water deficiency up-regulates the transcript levels of *TaPP2Ac-1* in wheat (*Triticum aestivum*) seedlings, and the overexpression of *TaPP2Ac-1* in tobacco (*Nicotiana tabacum*) enhances drought tolerance [47]. The expression of *TaPP2AbB"-a* is up-regulated by NaCl, polyethylene glycol (PEG), and cold and abscisic acid (ABA) stresses. Overexpression of *TaPP2AbB"-a* in *Arabidopsis* enhances lateral root development under NaCl and mannitol stresses [48]. PP2A has been shown to play a role in the chilling response in *Arabidopsis* in studies investigating two PP2A interactors: TAP46 and the AtCHIP E3 ubiquitin ligase [49, 50]. The *Arabidopsis RCN1* gene acts as an integrator of stress signaling and is a positive transducer of the response to ionic (Na^+^, K^+^), osmotic (mannitol), and oxidative (hydrogen peroxide) stress [36].

Many features of the functional analysis of PP2As reveal that they are key components of stress signaling transduction pathways, but whether they would respond to Pi deficiency remains unanswered. Moreover, knowledge of the responses of the RSA to Pi starvation in economically important crops is still limited because most studies have been conducted using *Arabidopsis* as a model system.

In this study, we produced *ZmPP2AA1* overexpression and RNAi (*ZmPP2AA1* OE and *ZmPP2AA1* RNAi) transgenic maize lines to discover the physiological functions of PP2A in the effects of Pi availability on the root system under different Pi regimens. The results showed that *ZmPP2AA1* was responsive to Pi stress. Relative to WT and *ZmPP2AA1* RNAi plants, *ZmPP2AA1* overexpressing plants presented inhibited and curly PRs with agravitropic growth, as well as increased LR density and LR length, independent of the Pi status of the plant. During Pi deprivation, LR and axial root (AR) formation was promoted in *ZmPP2AA1* overexpressing plants, which formed a highly branched root system that increased Pi acquisition under Pi deficiency. Overexpression of *ZmPP2AA1* modified the free IAA content in AR root tips and the sensitivity to exogenous IAA or NPA, which indicated that the regulation of *ZmPP2AA1* may be associated with auxin signaling. In addition, overexpression of *ZmPP2AA1* in maize enhanced Pi deficit stress tolerance and increased yields under Pi deficiency.

## Materials and methods

### Cloning and sequence analysis of *ZmPP2AA1*

Amino acid alignment of three PP2AA1 proteins from maize (*Zea mays* L.): ZmPP2AA1 (GRMZM2G 164352; protein ID: Q32SG2), GRMZM2G102858, GRMZM2G122135, and three PP2AA proteins from *Arabidopsis* (*Arabidopsis thaliana*) was performed using the CLUSTAL X 2.0 program [51] and GeneDoc 3.2 program [52]. The HEAT (*huntingtin*, elongation factor 3, A subunit of protein phosphatase 2A and TOR1) repeats were identified according to Perry and Kleckner [53]. The corresponding genomic sequences and structures were obtained using the EnsemblPlants browser (http://plants.ensembl.org/index.html). A phylogenetic tree was constructed using MEGA5 (http://www.megasoftware.net/mega5/mega.html) [54] with three aligned ZmPP2AA protein sequences; two rice PP2AA protein sequences: Osl_30535, Os09g0249700; one barley PP2AA protein sequence: MLOC_2967; and two *Brachypodium* PP2AA protein sequences: BRADI4G08720 and BRADI4G08790. The neighbor-joining (NJ) method was employed in the phylogenetic tree construction.

### Construction of *ZmPP2AA1* overexpressing and RNAi construct vectors in plants

The ORF and fragment for the *ZmPP2AA1* (GRMZM2G164352) overexpressing and RNAi vectors, respectively, were obtained by PCR using maize root cDNA from the inbred line Qi-319 as a template. The complete *ZmPP2AA1* ORF with *BamH*Ⅰ restriction sites at the 5' and 3' ends was amplified using the *ZmPP2AA1* OE primers (S1 Table), and the PCR product was inserted into the modified binary plant vector pCAMBIA3300-PUbi::MCS-Tnos-P35S::*bar* (pCUB). The pCUB contains a phosphinothricin acetyltranferase gene (*bar*) that confers resistance to the herbicide glufosinate ammonium.

For the *ZmPP2AA1* RNAi construct, a 403-bp fragment of the *ZmPP2AA1*-encoding sequence was amplified using *ZmPP2AA1-*specific primers (S1 Table). The amplified *ZmPP2AA1* fragment was inserted in the sense orientation with respect to *Sma*Ⅰ and *Spe*Ⅰ, and in the antisense orientation with respect to *BamH*Ⅰ and *Sac*Ⅰ on both sides of the rice intron in vector pTCK303. Subsequently, the hairpin structure was inserted into the pCUB vector to construct the RNAi plant expression vector pCAMBIA3300-pUbi::*zmpp2aa1*-Tnos-P35S::*bar*. The RNAi vector also contained a *bar* gene. The resultant plasmids were then introduced into *Agrobacterium tumefaciens* strain GV3101 using the freeze-thaw method.

### Maize transformation and regeneration

Maize transformation and regeneration were performed as previously described by Li *et al*. [55]. The plant material was the maize (*Zea mays* L.) elite inbred line Qi-319. The transformed plantlets were screened by spraying the herbicide Finale (contains 245 mg/l of glufosinate ammonium, produced by Aventis) at the 3-leaf stage, and the surviving seedlings were chosen for PCR. The herbicide-resistant and PCR-positive plants (T_0_) were transplanted in the field and self-pollinated for three generations. Every generation of transgenic plants was verified by herbicide selection and PCR.

### PCR analysis and Southern blotting

Genomic DNA from young maize leaves was extracted according to the cetyltrimethylammonium bromide (CTAB) method [56]. Detection of the *ZmPP2AA1* and *bar* genes was performed by PCR with *ZmPP2AA1-*specific and *bar* full-length primers, respectively (S1 Table). The PCR annealing temperatures were 52°C for the 621-bp *ZmPP2AA1* gene fragment and 56°C for the 560-bp *bar* gene fragment (S2 Fig). The genomic DNA isolated from the PCR-positive T_3_ transgenic maize leaves was digested with *Kpn*Ⅰ, and then Southern blotting was conducted using a digoxigenin (DIG)-labeled *ZmPP2AA1-*specific probe according to the DIG system manual (Roche).

### RNA isolation and qRT-PCR

Total RNA was extracted from plant samples using TRIzol reagent (TaKaRa). DNase-treated RNA was used as a template for cDNA synthesis using the RT reagent kit (TaKaRa) according to the manufacturer’s protocol. Real-time quantitative RT-PCR was performed using a LightCycler 480 (Roche) with the SYBR^®^ RT-PCR kit (TaKaRa) in 10-μL reaction volumes for each sample amplified through 40 cycles. The gene-specific primers *ZmPP2AA1* RT-F and *ZmPP2AA1* RT-R were used to detect *ZmPP2AA1* cDNA. The primers *actin* RT-F and *actin* RT-R (S1 Table) were designed to amplify the maize *actin* gene fragment as an internal control. The relative gene expression levels were calculated using the 2^-ΔΔCt^ method [57]. Each experiment was repeated three times.

### Hydroponic culture for the short-term experiment

The hydroponic culture and Pi deficiency treatments were established and maintained as described by Li et al. [58]. Seeds from Qi-319 and homozygous transgenic lines were surface-sterilized with 70% ethanol and 0.1% HgCl and washed with deionized water. They were then germinated on moist filter paper at 28°C in the dark for 3 d and transferred to a sufficient phosphate (SP, 1,000 μM KH_2_PO_4_) nutrient solution (2 mM Ca(NO_3_)_2_.4H_2_O, 1.25 mM NH_4_NO_3_, 0.1 mM KCl, 0.65 mM K_2_SO_4_, 0.65 mM MgSO_4_, 10 mM H_3_BO_3_, 0.5 mM (NH4)_6_Mo_7_O_24_, 1 mM MnSO_4_, 0.1 mM CuSO_4_.5H_2_O, 1 mM ZnSO_4_.7H_2_O, and 0.1 mM Fe-EDTA). At the 2-leaf stage (8 d old), the endosperms were carefully excised from all seedlings. After a recovery period of 2 d in the SP solution, half of the seedlings were transferred to the SP solution, and the remaining seedlings were grown in a low phosphate (LP, 5 μM KH_2_PO_4_) solution. Next, 1 mM KCl was added as a source of additional potassium to the LP treatment. The initial pH value of the nutrient solution was adjusted to 6.0±0.1. The nutrient solution was refreshed every 3 d. The plants grew under a 14-h light/10-h dark cycle at 32/25°C with 700 μmol m^-2^ s^-1^ photon flux density in a greenhouse with a relative humidity of approximately 65%. After treatment for 15 d, the plants were harvested to measure the biomass, morphological parameters, and total P content.

### Hormone treatment

The LP and SP nutrient solutions were supplemented with either 3 μM IAA or 3 μM NPA and cultured for 15 d.

### Long-term experiment

Seeds of the WT and transgenic plants were sown in cylindrical plastic pots containing loam, perlite, and roseite at a 2:1:1 ratio. After germination, the seedlings were irrigated with the LP (5 μM KH_2_PO_4_) nutrient solution every 2 d. At the 3-leaf stage, the plants were thinned to one plant per pot. When the plants reached the inflorescence stage, the anthesis-silking interval (ASI) was recorded, and only one ear was maintained on each maize plant. Mature ears were harvested to measure the ear length and kernel number per ear. After drying to a constant weight, the weight of the kernels was recorded to determine the yield.

### Quantification of biomass and P content

Root and shoot samples were harvested and dried at 80°C in an oven to a constant weight for biomass determination. Quantification of the total P content was performed using the method described by Murphy and Riley [59]. Approximately 200 mg of dried shoot or root samples were flamed to ash and dissolved in 4 ml of concentrated H_2_SO_4_ on an electric stove. The dissolved solution was diluted 10 times, and 0.5 ml of the diluted solution was added to 4 ml of coloration solution containing 3 mol/L H_2_SO_4_, 2.5% NH_4_M_0_O_4_, and 10% ascorbic acid. The absorbance was read at 660 nm. The P concentrations were expressed as mg P/g tissue DW, and the P contents were calculated by multiplying the P concentration by the DW, providing a value of mg P per plant.

### Estimation of Pi uptake kinetic parameters

The Pi uptake kinetics were assessed according to Drew and Saker [60]. The hydroponic-cultured maize seedlings were treated with SP (1,000 μM KH_2_PO_4_) and LP (5 μM KH_2_PO_4_) for 15 d, and all maize plants were transferred to the nutrient solution without P supplementation for 24 h. The P-depleted plants were transferred to the initial nutrient solution supplemented with 50 μM Pi. One milliliter of solution from each pot was collected at 30-minute intervals to measure the amount of Pi removed from the solution and to calculate the rate of Pi uptake. The Pi uptake kinetic parameters were estimated according to the method described by Claassen and Barber [61] using three replicates.

### Measurement of morphological parameters

For the sake of simplicity, primary roots, seminal roots and crown roots are referred to as ARs in this study. The number of ARs and LRs was counted according to Li et al. [9]. The PR length was measured with a ruler, and other root lengths were measured using the grid-line intersection method presented by Li et al. [9]. The total absorption area and effective absorption area of the roots were measured using the methyl blue method [62].

### Free IAA measurements

The IAA concentrations were measured in the 1.5-cm tips of the ARs of the WT and transgenic plants. The roots of plants at 15 d after treatment (DAT) to SP or LP were washed with deionized water five times for surface clearing. The root tips were excised and immediately frozen in liquid nitrogen. Two hundred milligrams of the pooled samples were analyzed using UPLC-MS/MS, i.e., a UPLC system (ACQUITY UPLC, Waters, Shanghai, China) and a triple quadrupole tandem mass spectrometer as described by Fu et al. [63] on the plant hormone measurement platform at the Institute of Genetics and Development Biology, Chinese Academy of Sciences.

### Statistical analysis

All data are presented as the mean values of at least three independent sets of experiments. Each value is expressed as the mean ± SD. Statistical significance between mean values was determined using one-way ANOVA with the SPSS 16.0 program. Different letters are used to indicate means that are significantly different at the 0.05 level.

## Results

### Cloning, sequence analysis, genomic organization and phylogenetic tree analysis of *ZmPP2AA1*

**Fig 1:**
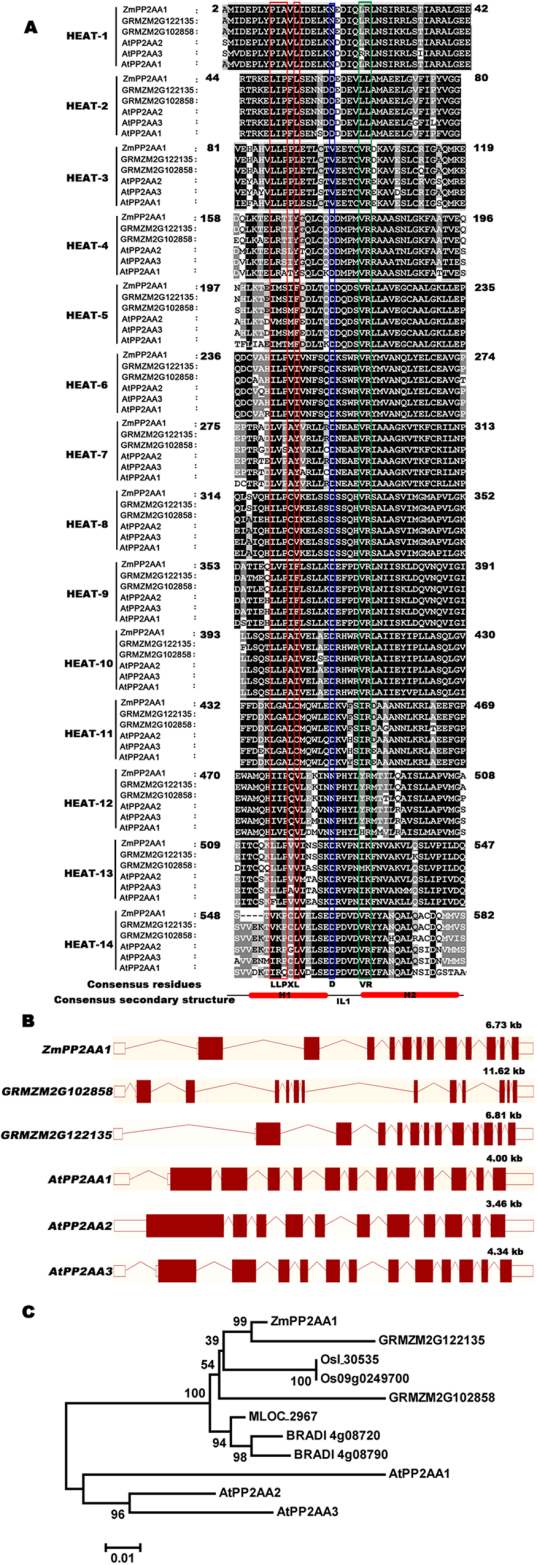
Alignment of HEAT repeats of ZmPP2AAs with AtPP2AAs, genomic organization of *ZmPP2AAs* and *AtPP2AA*s, and phylogenetic relationship analysis of PP2AAs between maize, *Arabidopsis*, rice, barley and *Brachypodium*. **(A)** Multiple sequence alignment of HEAT repeats in ZmPP2AAs with AtPP2AAs. The consensus “LLPXL” motifs in α-helix1 (H1) are boxed in red. The conserved Asp in the intra-loop (IL1) is boxed in blue. The consensus “VR” motifs in α-helix2 (H2) are boxed in green. Conserved residues between sequences are boxed in black or gray based on the degree of conservation. **(B)** Comparison of the genomic structures of *ZmPP2AAs* and *AtPP2AA*s. Exon/intron structures were obtained from the *EnsemblPlants* browser (http://plants.ensembl.org/index.html). Red boxes and lines denote protein coding regions and introns, respectively. (**C**) Neighbor-joining phylogenetic tree of PP2AAs. Bootstrap values are presented for all branches. Amino acid sequences for PP2AAs were obtained from http://www.uniprot.org: maize: ZmPP2AA1 (GRMZM2G164352), GRMZM2G102858, and GRMZM2G122135; rice: Osl_30535 and Os09g0249700; barley: MLOC_2967; *Brachypodium*: BRADI4G08720 and BRADI4G08790; *Arabidopsis*: AtPP2AA1 (AT1G25490), AtPP2AA2 (AT3G25800) and AtPP2AA3 (AT1G13320).

According to the mRNA sequence of the deduced *Zea mays* L. protein phosphatase 2A regulatory subunit A (Accession NO. AY940682), a 1765-bp fragment of the complete open reading frame (ORF) was produced by PCR from maize seedling root cDNA. This sequence was designated as *ZmPP2AA1*. The predicated protein of *ZmPP2AA1* comprises 583 amino-acid residues with a calculated molecular weight of 65 kDa and a theoretical isoelectric point of 4.93. The predicted ZmPP2AA1 protein shows 83.8%, 88.9%, and 87.6% amino acid identity with AtPP2AA1 (RCN1), AtPP2AA2, and AtPP2AA3, respectively (S1 Fig). The *ZmPP2AA1* genomic sequence spans 6.7 kb and contains 13 exons, of which 12 are coding exons. This profile is similar to those of *AtPP2AAs* (Fig 1B). *ZmPP2AA1* is located on chromosome 6 according to the maize genomic sequence at http://www.maizesequence.org/index.html. Another two ZmPP2AA homologs have also been found in maize. The deduced amino acid sequence of ZmPP2AA1 shares approximately 93% similarity with GMZM2G102858 and approximately 91% similarity with GMZM2G122135 (S1 Fig). All ZmPP2AA proteins contain classical “HEAT” repeats with similarity to AtPP2AAs (Fig 1A). The phylogenetic tree indicates that the PP2AA proteins from the grass family formed a major clade, while three AtPP2AA proteins formed a group (Fig 1C).

### *ZmPP2AA1* responds to Pi starvation

**Fig 2:**
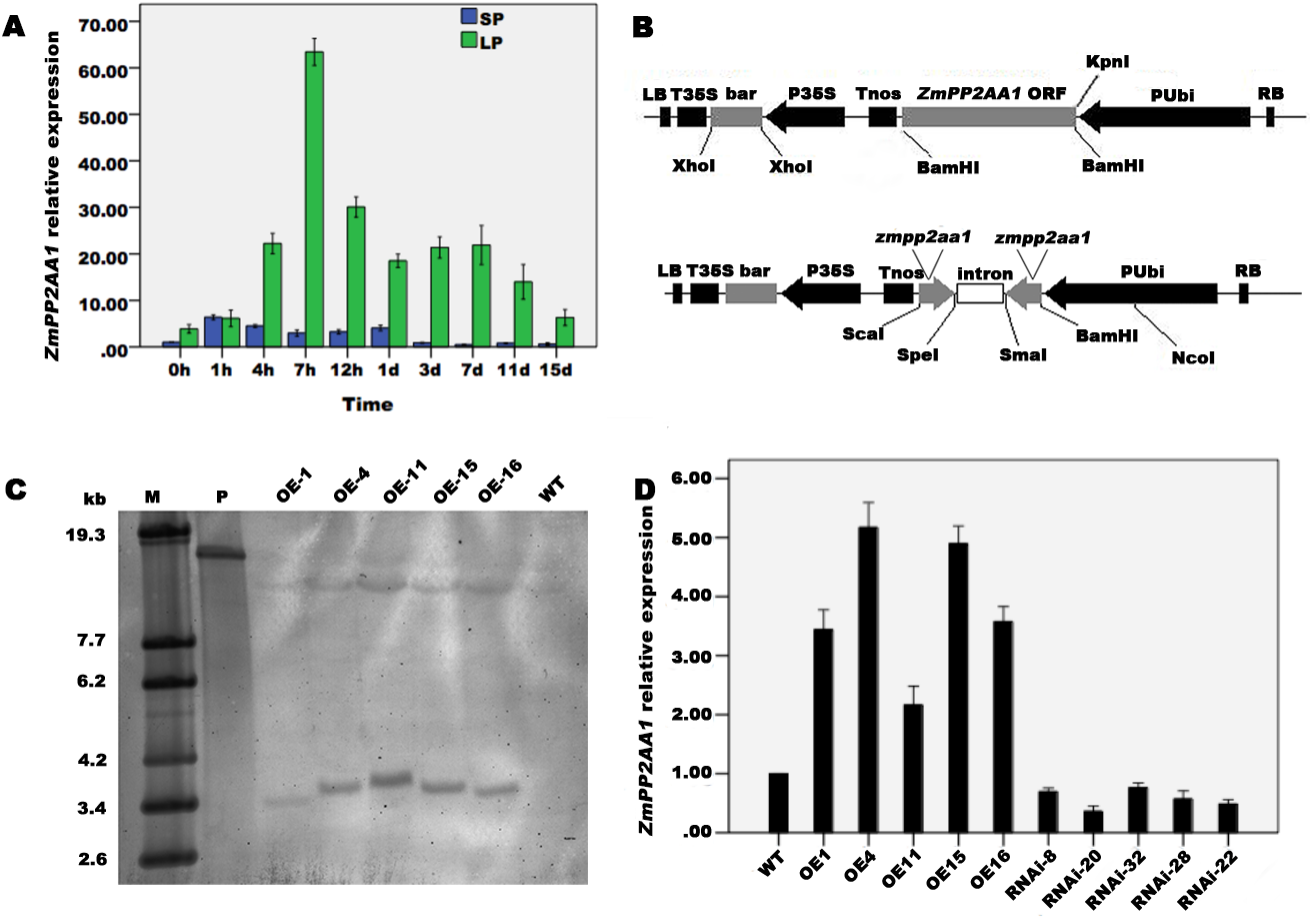
Expression pattern of *ZmPP2AA1* in roots and molecular characterization of *ZmPP2AA1* transgenic maize plants. **(A)** Expression pattern of *ZmPP2AA1* in roots cultured in SP (1,000 μM KH_2_PO_4_) and LP (5 μM KH_2_PO_4_) nutrient solutions. The expression of *ZmPP2AA1* was analyzed using qRT-PCR, and the value of the 0 h time point under the SP condition was considered 1-fold. **(B)** The T-DNA region of the vector used for the transformation. PUbi, a maize-constitutive ubiquitin promoter; *bar*, a biolaphos resistance phosphinothricin acetyl transferase gene; P35S, a promoter of CaMV35S from cauliflower mosaic virus. **(C)** Southern blotting analysis of the *ZmPP2AA1* gene. M, λ-DNA/*Eco*T14Ⅰ molecular weight marker; P, vector plasmid; OE-1, OE-4, OE-11, OE-15, OE-16, T_3_ transgenic plants overexpressing *ZmPP2AA1*; WT, wild-type Qi-319. **(D)** qRT-PCR analysis of *ZmPP2AA1* expression in the roots of maize transgenic lines and WT plants. The value of WT was considered 1-fold. Fold changes in the expression transcripts of all qRT-PCR analyses were calculated using the 2^-ΔΔCt^ method with maize *actin* as an internal control. The data represent the average of three independent experiments ± SD.

To assess the expression pattern of *ZmPP2AA1* in response to Pi starvation, the relative transcript abundance of *ZmPP2AA1* in the roots of the maize inbred line Qi-319 grown under Pi-deficient conditions was evaluated (Fig 2A). qRT-PCR revealed that the abundance of *ZmPP2AA1* transcripts in roots treated with LP was increased relative to the SP treatment. During early Pi deprivation (1 h ~7 h after treatment), the transcripts continually increased and reached a peak after 7 h, followed by a remarkable decrease in the transcript abundance at 12 h; however, the expression of *ZmPP2AA1* was maintained at a higher level after LP treatment compared with the SP treatment. The results indicate that *ZmPP2AA1* is a gene that responds to Pi deficiency with induced expression.

### Molecular characterization of *ZmPP2AA1* transgenic maize plants

To quantify the effect of *ZmPP2AA1* on plant responses to low Pi stress, we constructed transgenic plants with constitutive overexpression and RNA interference of *ZmPP2AA1* (Fig 2B) and transformed the inbred line Q319. The transgenic plants (T_0_ to T_2_ generation) were screened with respect to resistance to the herbicide Finale (glufosinate) and by PCR (S2 Fig). The herbicide-resistant and PCR-positive independent transgenic lines were further identified using Southern blotting analysis. As shown in Fig 2C, an endogenous *ZmPP2AA1* gene fragment was observed in all genotypes, while exogenous *ZmPP2AA1* was presented as specific hybridization patterns. These results suggest that the exogenous fragments were stably integrated into the maize genome.

qRT-PCR was conducted to analyze the transcript abundance of *ZmPP2AA1* in transgenic plants. Different expression levels were observed among different lines under normal conditions (Fig 2D). The overexpressing line OE-4 with a high transcription level (more than 5-fold higher than WT) and OE-11 with a low transcription level (2-fold higher than WT) among the overexpressing lines were chosen for further analysis. In RNAi plants, the presence of a few copies of *ZmPP2AA1* transcripts suggested that the expression of *ZmPP2AA1* was suppressed but not completely silenced. RNAi-20 with strong suppression (0.36-fold lower than WT) and RNAi-22 with mild suppression (0.48-fold lower than WT) were selected for subsequent characterization.

### Overexpression and suppression of *ZmPP2AA1* affect the development of the PR and LRs of maize

**Fig 3:**
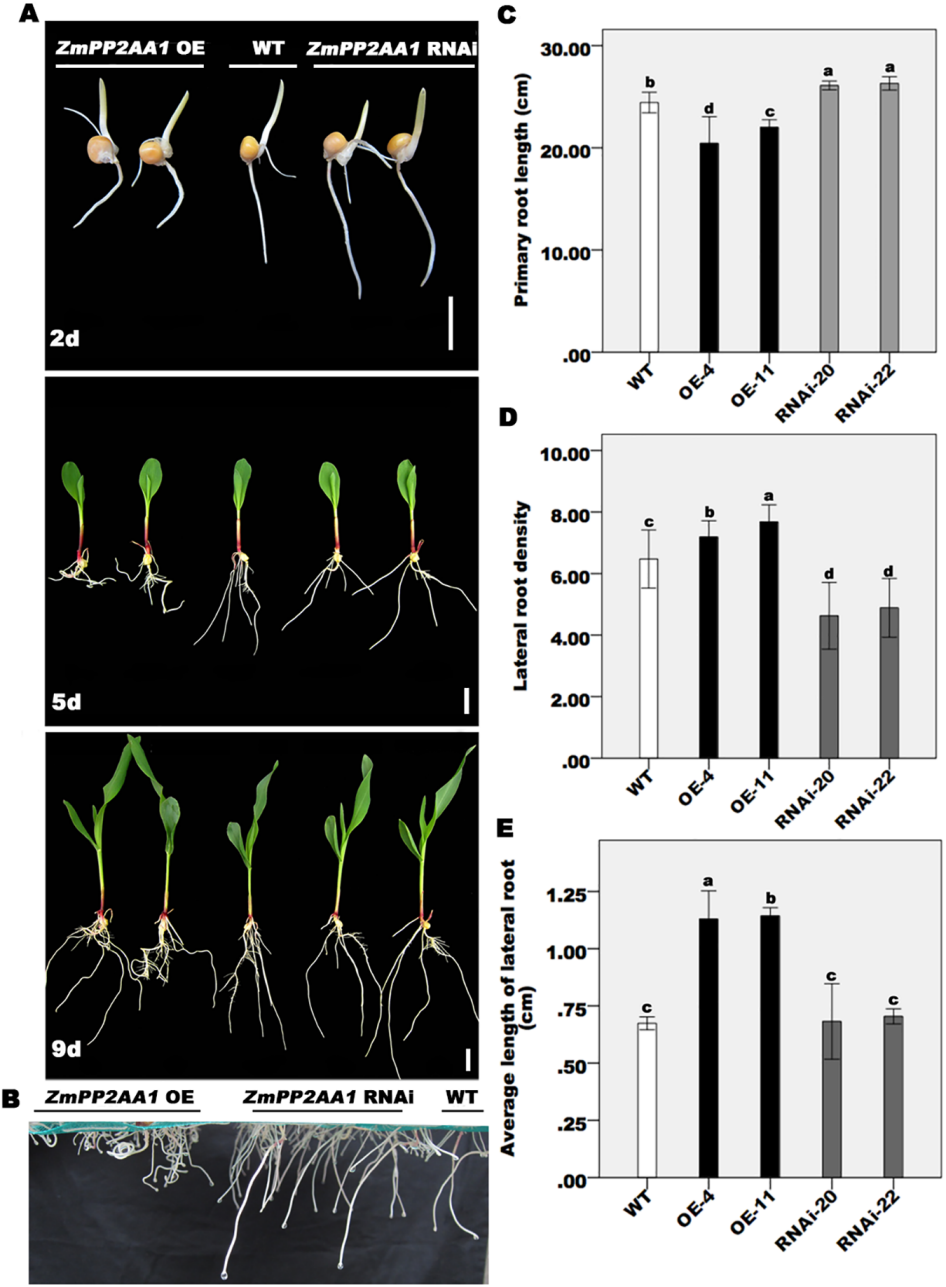
Modification of the expression levels of *ZmPP2AA1* in maize plants changes the root system architecture. (**A**) Photographs are of representative WT, *ZmPP2AA1* overexpressing and RNAi suppression transgenic seedlings grown in sufficient Pi (1,000 μM KH_2_PO_4_) nutrient solution for 2, 5 and 9 d after germination (DAG). (**B**) Root phenotype of the WT and *ZmPP2AA1* transgenic seedlings at 6 DAG under normal conditions. (**C, D, and E**) The PR length, LR density (LR number/cm PR) and average LR length of 15 DAG WT, *ZmPP2AA1* overexpressing plants and *ZmPP2AA1* RNAi plants. Values are the means ± SD of each genotype. Different letters on the bars indicate significant differences between the means (*P*< 0.05).

The *Arabidopsis* ROOTS CURL IN NAPHTHYLPHTHALAMIC ACID1 (*RCN1*) gene encodes the A1 isoform of the regulatory A subunit. The *Arabidopsis* mutant *rcn1* was isolated based on the presence of curled roots in the presence of the auxin transport inhibitor NPA, and the roots of mutant seedlings presented an abnormal curling growth pattern without NPA. The mutation in *RCN1* reduced the PR and hypocotyl elongation [41]. To determine whether the mutation in *ZmPP2AA1* could affect maize root and hypocotyl growth patterns, the phenotypes of the WT, *ZmPP2AA1* OE, and the *ZmPP2AA1* RNAi line were characterized in hydroponic culture. Hypocotyl elongation of *ZmPP2AA1* transgenic plants displayed a normal pattern. The roots of the *ZmPP2AA1* OE plants were curled, especially PR, which was not observed in the roots of the WT and *ZmPP2AA1* RNAi seedlings (Fig 3A and 3B; S2 Table). Compared with the WT, the PR length was reduced in the *ZmPP2AA1* OE plants, while that in the *ZmPP2AA1* RNAi lines was increased (Fig 3C; *P*< 0.05). The LR density and LR length of the *ZmPP2AA1* OE plants were significantly increased compared with the WT plants, while there was a significant decrease in the *ZmPP2AA1* RNAi lines (Fig 3D and 3E; *P*< 0.05). Together, the results suggest that *ZmPP2AA1* regulates PR growth and the gravitropic response, and it alters LR architecture by stimulating formation and elongation.

### *ZmPP2AA1* is involved in root system architecture remodeling in response to Pi starvation

**Fig 4:**
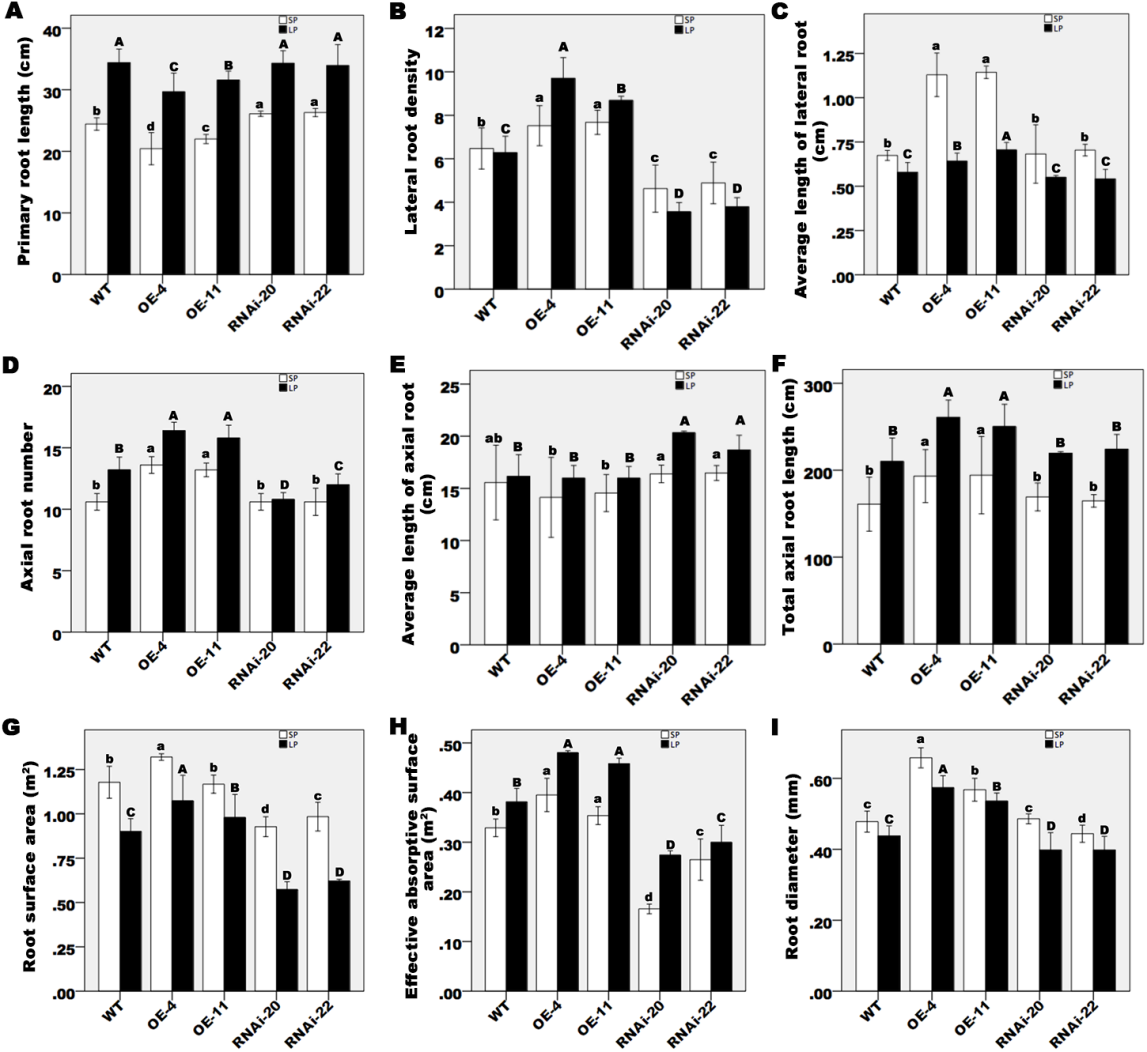
Root morphology of the WT and *ZmPP2AA1* transgenic plants in response to SP or LP treatment for 15 d. (**A**) PR length, (**B**) LR density (LR NO./cm PR), (**C**) average length of LR, (**D**) AR number, (**E**) average length of AR, (**F**) total length of AR, (**G**) root surface area, (**H**) effective absorptive surface area, and (**I**) root diameter of WT and transgenic plants hydroponically cultured under SP and LP conditions. Values are the means ± SD of each genotype. Different letters above the bars indicate significant differences between the means (*P*< 0.05) under the same conditions.

Modification of the RSA is a typical developmental response to Pi deficiency in the model eudicot *Arabidopsis* and in monocots such as rice and maize. An examination of the RSA response to Pi starvation in the variation of the expression level of *ZmPP2AA1* demonstrated that the variation could affect the development of the maize root system. After being subjected to SP or LP conditions for 15 d, the hydroponically grown WT and *ZmPP2AA1* transgenic plants were collected for root morphology analysis. The results showed that low Pi availability promoted PR and AR growth in all genotypes (Fig 4A, 4D, 4E and 4F). In addition, the average AR length in the *ZmPP2AA1* OE seedlings was comparable to WT, and the higher number of ARs resulted in longer total AR lengths in the OE lines under the LP condition. The down-regulation of *ZmPP2AA1* promoted AR elongation but had little effect on AR initiation. These results indicate that *ZmPP2AA1* plays an important role in the AR development response to Pi deficiency.

Modification of the expression of *ZmPP2AA1* in maize also influenced LR development under the LP condition. LP inhibited LR elongation in all lines (Fig 4C). In the *ZmPP2AA1* OE lines, the lateral root density was increased by LP stress, whereas a clear reduction was observed in the *ZmPP2AA1* RNAi lines (Fig 4B). A robust root system of the *ZmPP2AA1* OE seedlings was formed in comparison to the WT and RNAi lines (Fig 4I). Pi starvation significantly decreased the total root surface area, but the effective absorptive surface area increased in all lines (Fig 4G and 4H). In addition, compared with WT, the *ZmPP2AA1* OE seedlings exhibited a larger effective absorptive surface area, whereas a smaller area was observed in the RNAi lines. These results indicate that overexpression of *ZmPP2AA1* induced a highly branched root system with improved ARs and LRs in response to LP.

### Overexpression of *ZmPP2AA1* enhances tolerance to Pi deficiency in transgenic maize

After 11 d of Pi deficiency, the leaf veins and stems of the WT and *ZmPP2AA1* RNAi seedlings had accumulated anthocyanin, a typical Pi starvation response in plants [1], but the responses of the *ZmPP2AA1* OE lines were delayed. Compared with the seedlings grown under SP conditions, the DWs of the shoots of all seedlings grown under LP conditions were reduced, whereas the root DWs increased (Table 1). These results indicate that Pi deficiency favored root growth over shoot growth, which resulted in an increased root-to-shoot ratio of Pi-deprived plants compared with Pi-sufficient plants (Table 1). Under SP conditions, *ZmPP2AA1* overexpressing seedlings exhibited comparable root and shoot biomasses to the WT plants but an 18% higher root biomass and a 12% higher shoot biomass than the WT plants under LP conditions. These findings suggest that the overexpression of *ZmPP2AA1* led to delayed anthocyanin accumulation and resulted in a much higher biomass under Pi starvation.

**Table 1:** Biomass of WT and transgenic plants under SP and LP conditions.

Dry weight (g)	WT	OE-4	OE-11	RNAi-20	RNAi-22
Root						
SP	0.14 ± 0.01 a	0.14 ± 0.01 a	0.14 ± 0.01 a	0.08 ± 0.01 b	0.08 ± 0.01 b
LP	0.17 ± 0.01 b	0.20 ± 0.01 a	0.19 ± 0.01 a	0.15 ± 0.01 c	0.16 ± 0.01 bc
Shoot						
SP	0.67 ± 0.01 b	0.68 ± 0.02 ab	0.70 ± 0.01 a	0.49 ± 0.01 c	0.49 ± 0.02 c
LP	0.52 ± 0.01 b	0.58 ± 0.02 a	0.57 ± 0.01 a	0.37 ± 0.01 d	0.40 ± 0.01 c
Root/shoot						
SP	0.21 ± 0.02 a	0.20 ± 0.01 a	0.19 ± 0.01 a	0.16 ± 0.02 b	0.17 ± 0.02 b
LP	0.32 ± 0.01 c	0.35 ± 0.02 b	0.33 ± 0.02 bc	0.41 ± 0.02 a	0.40 ± 0.03 a

The DWs of the roots and shoots of the WT, overexpressing (OE-4 and OE-11), and RNAi (RNAi-20 and RNAi-22) plants were determined after drying to a constant weight in an oven at 80°C. Values presented are the means ± SD (n = 5). Values followed by different letters indicate significant differences (*P*< 0.05) among seedlings grown under the same Pi conditions.

### Total P accumulation increases in *ZmPP2AA1* overexpressing transgenic maize plants during Pi deprivation

**Fig 5:**
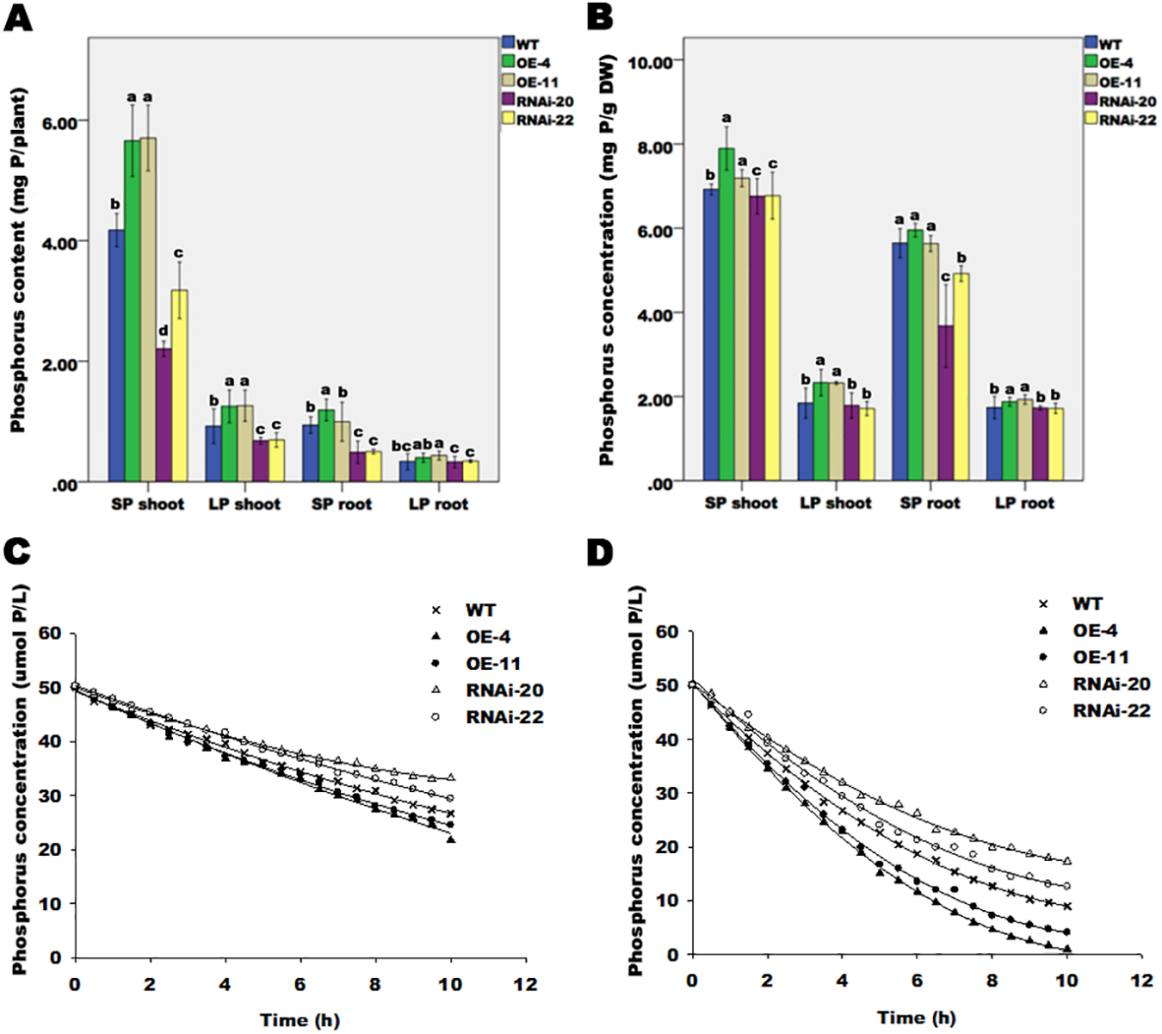
Enhanced Pi absorptive capacity enables *ZmPP2AA1* OE to accumulate more phosphorus than the WT and *ZmPP2AA1* RNAi plants. WT and transgenic plants were grown under SP or LP conditions for 15 d. Phosphorus content **(A)** and phosphorus concentration **(B)** in WT and transgenic plants. Values represent the means ± SD. Different letters above the bars are used to indicate significant differences (*P*< 0.05) among means under the same conditions. **(C, D)** Pi uptake kinetics in WT and transgenic plants under SP and LP conditions. The maize seedlings were subjected to SP (**C**) and LP (**D**) conditions for 15 d and were then transferred to Pi starvation conditions without P supplementation for 24 h. The P-depleted plants were transferred to the initial nutrient solution supplemented with 50 μM Pi for the depletion experiment. Pi uptake was measured as the Pi removed from the nutrient solution over time. The points represent observed values. The curve was fitted using an expression based on Michaelis-Menten kinetics.

To address the effect of *ZmPP2AA1* on Pi homeostasis, we measured the total P contents (mg P/plant) and P concentrations (mg P/g DW) in WT and transgenic plants grown under SP or LP treatment for 15 d (Fig 5A and 5B). Under SP conditions, OE-4 and OE-11 had 35% and 37% higher shoot P contents, respectively, than WT. In contrast, RNAi-20 and RNAi-22 had 47% and 24% lower shoot P contents, respectively, compared with WT. OE-4 had the highest root P content (1.19 mg P), while RNAi-20 had the lowest root P content (0.49 mg P). Under Pi-deficient conditions, the shoot and root P contents decreased in all plants. In comparison to WT, *ZmPP2AA1* OE exhibited a 36% higher average shoot P content, whereas a reduction of 22% was observed in *ZmPP2AA1* RNAi.

We calculated the P concentration as the total P content normalized to the biomass (mg P/g DW) (Fig 5B). The down-regulation of *ZmPP2AA1* significantly reduced (*P*<0.05) the shoot P concentrations compared with the WT and *ZmPP2AA1* OE plants under both SP and LP conditions (Fig 5B). There were no significant differences (*P*> 0.05) in root P concentrations among the WT and overexpressing lines under SP conditions, but under LP conditions, the *ZmPP2AA1* OE seedlings had significantly higher P concentrations (*P*< 0.05) in the shoots and roots compared with the WT. These results suggest that plants overexpressing *ZmPP2AA1* with highly branched root systems have higher phosphate acquisition capability and accumulate more P in response to LP treatment. The maintenance of higher P concentrations could help alleviate plant growth retardation under Pi stress.

### Different P uptake capacities of *ZmPP2AA1* overexpressing and RNAi transgenic maize plants

To determine whether the variations among genotypes with respect to the total P contents and concentrations were driven by differences in P influx, we measured the P uptake kinetics. Examples of the results obtained for the plants subjected to SP and LP conditions in the depletion experiments are shown in Fig 5C and Fig 5D, respectively. The rate of depletion in plants grown in the SP solution (Fig 5C) was relatively gradual in comparison with plants grown in the LP solution (Fig 5D). *ZmPP2AA1* OE displayed a steeper slope compared with the other plants, reflecting a higher rate of P uptake (Fig 5C and 5D). Table 2 shows the Pi uptake parameters for the WT and transgenic plants grown in the presence of different Pi levels. In comparison to the plants subjected to SP conditions, the Pi uptake capability of all plants subjected to LP conditions increased remarkably, as indicated by their increased maximal P uptake rate (*Imax*), lower Michaelis-Menten constant (*Km*) and lower minimal concentration (*Cmin*) (Table 2). Differences in Pi uptake parameters were also observed among the WT and transgenic plants. Regardless of Pi treatment, the *Imax* values of *ZmPP2AA1* OE were higher whereas those of *ZmPP2AA1* RNAi were lower than the WT. Compared with the WT, the *ZmPP2AA1* OE seedlings exhibited significantly lower *Km* and *Cmin* parameters whereas the *ZmPP2AA1* RNAi seedlings exhibited remarkably higher values (Table 2, *P*< 0.05) under LP conditions. These data indicate an enhanced rate of Pi uptake in the *ZmPP2AA1* OE plants, which might explain the increased accumulation of total P in the overexpressing plants.

**Table 2:** Pi uptake kinetics in WT and transgenic plants under SP and LP conditions.

	WT	OE-4	OE-11	RNAi-20	RNAi-22
*I*_*ma*x_ (μmol l^-1^ h^-1^)					
SP	2.80 ± 0.05 c	3.31 ± 0.05 a	3.15 ± 0.02 b	2.55 ± 0.03 d	2.54 ± 0.01 d
LP	7.00 ± 0.02 c	8.78 ± 0.03 a	8.20 ± 0.12 b	5.48 ± 0.02 e	6.58 ± 0.07 d
*C*_*mi*n_ (μmol l^-1^)					
SP	12.08 ± 1.38 c	12.11 ± 0.54 c	12.83 ± 1.15 c	29.81 ± 0.45 a	23.62 ± 2.03 b
LP	7.76 ± 1.02 c	1.83 ± 0.14 e	3.24 ± 0.44 d	15.5 ± 0.35 a	12.32 ± 1.03 b
*K*_*m*_ (μmol l^-1^)					
SP	21.41 ± 1.06 c	21.52 ± 0.42 c	21.97 ± 0.87 c	34.89 ± 0.30 a	29.82 ± 2.73 b
LP	18.36 ± 0.69 c	14.02 ± 0.08 d	15.04 ± 0.31 d	24.20 ± 0.39 a	20.01 ± 1.13 b

The maize seedlings were subjected to SP and LP conditions for 15 d and were then transferred to Pi starvation conditions without P supplementation for 24 h. The P-depleted plants were transferred to the initial nutrient solution supplemented with 50 μM Pi for the depletion experiment. The Pi parameters were estimated according to the method described by Claassen and Barber [61]. *Imax*, maximal P uptake rate; *Cmin*, minimal concentration below which no further net influx occurred; *Km*, Michaelis-Menten constant. Values are the means ± SD (n = 3). Values followed by different letters indicate significant differences at the 0.05 level among different genotypes under the same Pi concentrations. WT, wild-type; OE-4 and OE-11, overexpressing lines; RNAi-20 and RNAi-22, RNA interference lines.

### *ZmPP2AA1* overexpressing maize plants produce higher yields than WT and RNAi plants under Pi starvation conditions

We used cylindrical pots with a low Pi concentration to assess the influence of Pi deficiency on inflorescence and kernel development of the maize plants. Compared with the overexpressing lines, a significantly longer anthesis-silking interval (ASI) was observed in the WT and RNAi plants (Table 3, *P*< 0.05). More tassel branches were found on the *ZmPP2AA1* OE plants compared with the WT and *ZmPP2AA1* RNAi lines (Table 3). The yields of the plants are shown in Table 3. Compared with the WT, the ear weights of OE-4 and OE-11 increased by 45% and 26%, respectively, due to their larger kernels (higher 100-grain weights) and higher number of kernels per ear.

**Table 3:** Agronomic traits of WT and transgenic plants under Pi-deficient conditions.

	WT	OE-4	OE-11	RNAi-20	RNAi-22
Number of tassel branches	5.67 ± 0.58 b	7.67 ± 0.58 a	7.00 ± 0.58 a	5.00 ± 0.58 b	5.33 ± 0.58 b
ASI (days)	3.67 ± 0.58 a	1.67 ± 0.58 b	1.33 ± 0.58 b	4.33 ± 0.58 a	4.00 ± 1.00 a
Ear length (cm)	9.87 ± 0.40 c	14.23 ± 0.81 a	12.87 ± 0.60 b	8.63 ± 0.49 d	8.87 ± 0.25 d
Ear weight (g)	34.87 ± 1.70 c	50.47 ± 1.11 a	43.83 ± 1.63 b	30.30 ± 1.01 d	31.90 ± 0.70 d
Grain number per ear	235.00 ± 3.61 c	262.67 ± 5.51 a	250.33 ± 4.16 b	230.00 ± 4.58 c	232.00 ± 3.46 c
100-grain weight (g)	13.09 ± 0.26 c	17.35 ± 0.30 a	15.65 ± 0.26 b	11.36 ± 0.27 d	11.72 ± 0.26 d

Plants were grown in cylindrical pots with LP (5 μM KH_2_PO_4_) supplementation. At the flowering stage, the tassel branches and ASI were determined. After harvesting, the ear length and grain number per ear were recorded, and dried ears were weighed to determine the kernel yields. Values are the means ± SD (n = 5). Values followed by different letters indicate significant differences at the 0.05 level between the WT and transgenic lines under LP conditions. WT, wild-type; OE-4 and OE-11, overexpressing lines; RNAi-20 and RNAi-22, RNAi lines.

### Free IAA contents in the PR of *ZmPP2AA1* OE are higher than those in WT and *ZmPP2AA1* RNAi under SP but not LP conditions

Auxin plays a key role in root development and mediates the Pi starvation effects on the RSA [2, 15, 16, 18–20]. To examine whether the curly and inhibited PRs of the *ZmPP2AA1* OE lines and the promotion of AR elongation in the seedlings grown under LP conditions were affected by the variation in auxin accumulation in roots, we measured the free IAA contents in the apical 1.5 cm of the AR tips of seedlings grown under SP or LP conditions (Table 4). The root tips of the *ZmPP2AA1* OE seedlings with short and curly ARs accumulated more free auxin than the other two maize seedling genotypes (Table 4). This indicated that the changes in AR development might be attributable to the variation in auxin levels. Pi-starved WT and *ZmPP2AA1* RNAi seedlings exhibited comparable concentrations of free IAA to those measured in the Pi-sufficient seedlings. By contrast, the root tips of the *ZmPP2AA1* OE line had lower auxin levels under LP conditions.

**Table 4:** Free IAA content of WT and *ZmPP2AA1* transgenic plants under SP and LP conditions.

pg free IAA mg^-1^ FW			
	WT	*ZmPP2AA1* OE	*ZmPP2AA1* RNAi
SP	20.22 ± 1.34 b	27.54 ± 2.16 a	20.64 ± 2.02 b
LP	18.26 ± 1.70 b	21.38 ± 2.19 a	19.58 ± 1.65 b

Q319 (WT), *ZmPP2AA1* overexpressing line OE-4 and RNA interference line RNAi-20 were grown in SP (1,000 μM KH_2_PO_4_) or LP (5 μM KH_2_PO_4_) nutrient solution for 15 d. The apical 1.5-cm-long root tips of all plants were excised, and the free IAA content was determined in 200-mg pooled samples. Values are the means of three independent experiments. Values followed by different letters indicate significant differences at the 0.05 level under the same conditions. FW, fresh weight.

### *ZmPP2AA1* regulates an auxin response or auxin transport that is linked to root architecture remodeling under Pi deficiency

**Fig 6:**
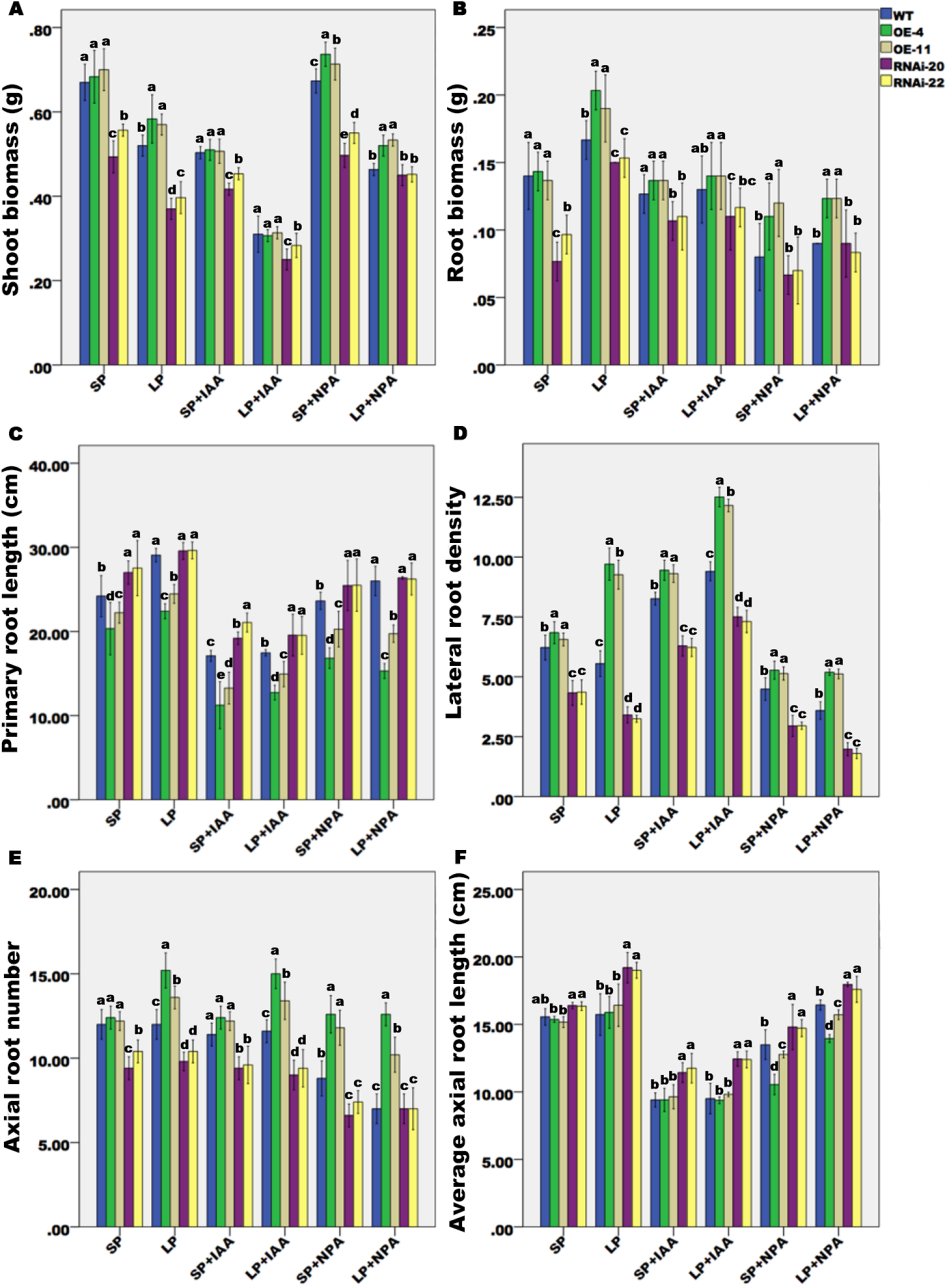
WT and *ZmPP2AA1* transgenic plants grown under SP or LP conditions respond differently to IAA and NPA. WT, overexpressing and RNAi plants were cultured under SP or LP conditions with or without IAA or NPA for 15 d. **(A**, **B)** Shoot and root biomasses of the WT and transgenic plants grown under SP or LP conditions with or without IAA or NPA for 15 d. **(C)** Primary root length, (**D**) LR density, (**E**) AR number, and (**F**) average AR length of WT and transgenic seedlings subjected to control conditions or treated with IAA or NPA. Values represent the means ± SD. Different letters above the bars indicate significant differences (*P*< 0.05) between the means under the same conditions.

To determine the relationship between LP signaling and auxin sensitivity or auxin transport in *ZmPP2AA1* OE, we analyzed the effect of IAA and NPA on maize RSA under LP and SP conditions (S3 Fig; Fig 6). The concentrations of applied IAA and NPA (both 3 μM) were determined based on preliminary work conducted using plants cultivated under a wide range of IAA and NPA levels. Exogenous IAA amplified the detrimental effect of Pi starvation on shoot growth (S3 Fig). Compared with SP+IAA, the shoot biomass decreased by 40% in response to the LP+IAA treatments (Fig 6A). IAA treatment (3 μM) hindered PR elongation and resulted in a shortened but thickened AR (S3 Fig; Fig 6C and 6F). The reduction in length was more substantial under LP+IAA conditions, demonstrating that IAA treatment suppressed or attenuated the positive effects of Pi deprivation on PR and AR elongation, leading to comparable root biomasses in the SP+IAA and LP+IAA seedlings (Fig 6B). The effects of IAA on the roots were similar in the WT and transgenic seedlings under SP or LP conditions.

NPA-treated seedlings exhibited suppressed root growth and a similar shoot biomass to the non-treated control (S3 Fig; Fig 6A and 6B). NPA treatment enhanced the agravitropic phenotype of the *ZmPP2AA1* OE lines, while both the WT and RNAi lines displayed a weak response (S3 Fig). In the preliminary experiment, 1 μM NPA resulted in apparent curving of the roots of the *ZmPP2AA1* OE seedlings, whereas a root agravitropic phenotype was observed in the WT plants exposed to up to 3 μM NPA. The stimulatory effect of LP on PR growth was eliminated by NPA treatment, and the detrimental effects were more drastic in *ZmPP2AA1* OE (Fig 6C). These results suggest that the overexpressing lines were more sensitive to the effect of the auxin transport inhibitor NPA on embryonic PR development, and the RNAi lines were not susceptible.

After 15 d of NPA treatment, the average AR length was barely affected (Fig 6F), and the numbers of ARs in the WT and *ZmPP2AA1* RNAi lines were strikingly reduced compared with the NPA controls under SP or LP conditions. However, the overexpressing lines were less sensitive to the effect of NPA treatment on AR numbers (Fig 6E). NPA decreased the number of LRs in all genotypes, and the LP+NPA treatment had stronger inhibitory effects on the RNAi lines compared with the SP+NPA treatment (Fig 6D).

## Discussion

In the present study, we found that *ZmPP2AA1* participated in the regulation of maize root development and played important roles in the response of maize seedlings to low phosphate stress. ZmPP2AA1 negatively regulated PR elongation and was involved in root gravitropism and LR development. Overexpression of *ZmPP2AA1* in maize plants resulted in a highly branched root system, maintained excellent shoot growth, increased Pi uptake and P content in roots, and increased grain yield under Pi deficit conditions. These results indicate that *ZmPP2AA1* overexpressing maize plants had greater tolerance for low phosphate conditions than the WT and *ZmPP2AA1* RNAi maize plants.

A high level of root branching has been reported to favor phosphorus uptake under conditions of low Pi availability [3, 64]. Compared with the WT and RNAi lines, the *ZmPP2AA1* OE plants showed enhanced AR and LR formation. An extensive root system with a larger absorptive area enhances exploitation in the upper layer of soil that contains Pi nutrient-rich regions (Fig 4). Efficient P uptake kinetics is another factor accounting for the high Pi uptake under low Pi supply (Fig 5C and 5D; Table 2). Higher *Imax* and lower *Km* and *Cmin* values indicated that the *ZmPP2AA1* OE lines had efficient Pi uptake (Table 2), which was related to the higher Pi contents and concentrations (Fig 5A and 5B).

Auxin plays a key role in maize root development [65–72]. It has been suggested that PP2A activity is required for the normal regulation of auxin transport in *Arabidopsis* [39]. Several auxin-regulated characteristics, such as the PR gravitropic response, PR elongation, and LR development, were altered in the *ZmPP2AA1* transgenic plants (Fig 3A–3E). Excessive free IAA in the *ZmPP2AA1* OE lines (Table 4) and the different responses of the WT and *ZmPP2AA1* transgenic plants to IAA and NPA (Fig 6) suggest that the regulation of ZmPP2AA1 in root development may be associated with auxin signaling.

Interestingly, the inhibited and curly PR roots were characterized as a result of the overexpression of *ZmPP2AA1* in maize (Fig 3), while the similar PR phenotype in *Arabidopsis* was caused by loss-of-function of *RCN1* [41]. The ZmPP2AA1 proteins exhibited a high amino acid sequence identity with AtPP2AAs and high conservation in the “HEAT” repeats (Fig 1A). The discrepant effects of PP2AA on root curling between maize and *Arabidopsis* may be attributed to the following: different responses to growth conditions [41]; differences in the roots of the dicotyledon *Arabidopsis* and monocot maize [8,10]; or differences in auxin distribution and accumulation patterns [27,28]. The *ZmPP2AA1* RNAi lines showed a relative weak phenotype, which may have occurred because RNA interference did not silence but did suppress *ZmPP2AA1*. ZmPP2AAs share high similarity in their amino acid sequences and are conserved in “HEAT” repeats, suggesting that *ZmPP2AA1*, one member of the PP2AA gene family, might have some functional redundancy with other members.

Several reports have indicated that PP2A responds to Pi starvation. An investigation of the transcriptional response of maize roots to Pi starvation revealed that one gene encoding the PP2AB’ kappa subunit (ID: Zm.85150) was up-regulated by low Pi availability [73]. Our qRT-PCR results showed that *ZmPP2AA1* was significantly up-regulated by low Pi availability (Fig 2A). The phosphoproteome profiles of maize ARs under LP stress also revealed that the catalytic subunit isoform 2 of PP2A displayed dynamic temporal patterns induced by low Pi availability [58]. This phenomenon suggests that the protein phosphatase ZmPP2AA1 is involved in a complex that plays a role in Pi starvation signal transduction following the perception of a low Pi signal.

Modified RSA in response to low Pi availability is often considered to be an adaptive response to maximize the Pi uptake capacity of plants in low Pi environments [2, 7, 15, 74–77]. A shortened PR is one of the characteristics of the altered the RSA induced by Pi starvation in *Arabidopsis* seedlings [6]. By contrast, in the present study, AR elongation was promoted in maize seedlings by Pi deficiency (Fig 4F), which is consistent with other reports [9, 58]. Pi deficiency inhibited LR elongation in all lines (Fig 4C). Low Pi availability increased LR density in the *ZmPP2AA1* OE lines but reduced it in the WT and *ZmPP2AA1* RNAi plants (Fig 4B). IAA or NPA treatment altered the effects of LP on ARs and LRs (Fig 6D–6F), suggesting that auxin may play a role in the effect of LP-induced root modification. However, *ZmPP2AA1* OE or *ZmPP2AA1* RNAi scarcely showed differences with the WT in the response of roots to Pi deficiency or the hormone treatments, except for LR density. These findings suggest that other factors or genes co-participate in the RSA response to LP.

The *ZmPP2AA1* overexpressing transgenic seedlings grown under Pi deficit conditions formed a highly branched root system, which enhanced the absorptive capability of Pi uptake. In addition, a significantly higher dry weight, root-to-shoot ratio, total P content and concentration, and delayed and reduced accumulation of anthocyanin was observed in these *ZmPP2AA1* overexpressing transgenic seedlings compared with the WT and *ZmPP2AA1* RNAi lines. The results suggest that the overall growth and development of the *ZmPP2AA1* OE lines indicated less Pi starvation stress.

## Conclusions

Based on the phenotypes of the transgenic plants overexpressing *ZmPP2AA1* or those with RNAi interference and their non-transgenic controls, it is concluded that *ZmPP2AA1* is a key player in PR growth and postembryonic root development. Auxin appears to be involved in the physiological processes underlying AR and LR development of maize under Pi deficiency. This research provides information on the low nutrient availability signal and auxin, as well as root development responses to LP conditions. The superior performance of plants with *ZmPP2AA1* overexpression under low Pi stress supports the application of *ZmPP2AA1* overexpression in the engineering of maize with improved tolerance to low phosphate conditions.

## Supporting information

S1 Table PCR primers for *ZmPP2AA1* cloning and molecular characterization of *ZmPP2AA1* transgenic maize. *ZmPP2AA1* ORF primers were used for full ORF amplification of *ZmPP2AA1* ORF; *ZmPP2AA1* OE and *ZmPP2AA1* RNAi primers were used for overexpression and RNAi vector construction, respectively; *ZmPP2AA1* PCR and *bar* PCR primers were used for *ZmPP2AA1* and *bar* detection in transgenic plants, respectively; *ZmPP2AA1* qRT and *actin* qRT primers were used for qRT-PCR for *ZmPP2AA1* and *actin*, respectively.
(PDF)

S2 Table Overexpression of *ZmPP2AA1* causes PR curling in maize. WT and transgenic seedlings were grown under SP or LP conditions for 6 days. Seedlings exhibiting root curling were counted. Values are the means ± SD (n = 20). The experiment was repeated five times.
(PDF)

S1 Fig Alignment of the deduced amino acid sequence of PP2AAs between maize, *Arabidopsis*, rice, barley and *Brachypodium*. Conserved residues between sequences are boxed in black or gray based on the degree of conservation. *Arabidopsis*: AtPP2AA1 (AT1G25490), AtPP2AA2 (AT3G25800) and AtPP2AA3 (AT1G13320); maize: ZmPP2AA1 (GRMZM2G164352), GRMZM2G102858, and GRMZM2G122135; rice: Osl_30535 and Os09g0249700; barley: MLOC_2967; *Brachypodium*: BRADI4G08720 and BRADI4G08790.
(PDF)

S2 Fig PCR analysis of transgenic plants. **(A)** PCR analysis of the *ZmPP2AA1* overexpressing T_3_ transgenic plants. M, DNA marker DL2,000; +, the PCR product of plasmid pCAMBIA3300-PUbi::*ZmPP2AA1*-Tnos-P35S::*bar*; -, the PCR product of H_2_O as a negative control template; WT, untransformed control Qi-319; OE-1, OE-4, OE-11, OE-15, OE-16, different *ZmPP2AA1* overexpressing transgenic lines. **(B)** PCR analysis of *ZmPP2AA1* RNAi T_3_ transgenic plants for the *bar* gene. M, DNA marker DL2,000; +, the PCR product of plasmid pCAMBIA3300-PUbi::*zmpp2aa1*-Tnos-P35S::*bar* as a positive control; WT, untransformed control Qi-319; RNAi-8, RNAi-10, RNAi-20, RNAi-22, RNAi-28, RNAi-32, different *ZmPP2AA1* RNAi transgenic lines.
(PDF)

S3 Fig Representative seedlings grown for 15 d under SP (left) or LP (right) solutions with or without IAA or NPA. Bar = 5 cm.
(PDF)
