# Supplementary material for: Overexpression of the protein phosphatase 2A regulatory subunit a gene ZmPP2AA1 improves low phosphate tolerance by remodeling the root system architecture of maize
Source: PLoS One. 2017 Apr 27;12(4):e0176538. doi: 10.1371/journal.pone.0176538 (PMC5407761; doi:10.1371/journal.pone.0176538)
Supplement: S1 Table — ZmPP2AA1 ORF primers were used for full ORF amplification of ZmPP2AA1 ORF; ZmPP2AA1 OE and ZmPP2AA1 RNAi primers were used for overexpression and RNAi vector construction, respectively; ZmPP2AA1 PCR and bar PCR primers were used for ZmPP2AA1 and bar detection in transgenic plants, respectively; ZmPP2AA1 qRT and actin qRT primers were used for qRT-PCR for ZmPP2AA1 and actin, respectively. (PDF) [file pone.0176538.s001.pdf]

S1 Table. PCR primers for *ZmPP2AA1* cloning and molecular characterization of *ZmPP2AA1* transgenic maize.

Primer	Sequence
ZmPP2AA1 ORF-F	CTGATGGCTATGATTGATG
ZmPP2AA1 ORF-R	CAAAGGCTAGTTAGCTTGA
ZmPP2AA1 OE-F	TTAGGATCCATGGCTATGATTGATG
ZmPP2AA1 OE-R	AAAGGATCCTTAGCTTGACACCATC
ZmPP2AA1 RNAi-F	CAACCCGGGACTAGTTGTGAGGCTGTTGGCCCT
ZmPP2AA1 RNAi-R	CTCGGATCCGAGCTCAATTGCTGGTAGCAGTGA
ZmPP2AA1 PCR-F	GCGTATCGTCCTGTGGTC
ZmPP2AA1 PCR-R	CAGGAGCCATTCCCATAA
bar PCR-F	ATGAGCCCAGAACGACGCC
bar PCR-R	TCAAATCTCGGTGACGGGC
ZmPP2AA1 qRT-F	GGCTGTGCTGCCCTTGGTA
ZmPP2AA1 qRT-R	CCTATCCGCACTTCAGCCTC
actin qRT-F	ATCACCATTGGGTCAGAAAGG
actin qRT-R	GTGCTGAGAGAAGCCAAAATAGAG

ZmPP2AA1 ORF primers were used for full ORF amplification of *ZmPP2AA1* ORF; *ZmPP2AA1* OE and *ZmPP2AA1* RNAi primers were used for overexpression and RNAi vectors construction, respectively; *ZmPP2AA1* PCR and *bar* PCR primers were used for *ZmPP2AA1* and *bar* detection in transgenic plants, respectively; *ZmPP2AA1* qRT and *actin* qRT primers were used for qRT-PCR for *ZmPP2AA1* and *actin*, respectively.
