# Supplementary material for: Overexpression of the protein phosphatase 2A regulatory subunit a gene ZmPP2AA1 improves low phosphate tolerance by remodeling the root system architecture of maize
Source: PLoS One. 2017 Apr 27;12(4):e0176538. doi: 10.1371/journal.pone.0176538 (PMC5407761; doi:10.1371/journal.pone.0176538)
Supplement: S2 Table — WT and transgenic seedlings were grown under SP or LP conditions for 6 days. Seedlings exhibiting root curling were counted. Values are the means ± SD (n = 20). The experiment was repeated five times. (PDF) [file pone.0176538.s002.pdf]

S2 Table. Overexpression of *ZmPP2AA1* causes PR curling in maize.

Conditions	No. of seedlings exhibiting curling root				
	WT	OE-4	OE-11	RNAi-20	RNAi-22
SP	1.20 ± 0.84 b	17.8 ± 0.84 a	17.8 ± 0.84 a	0.8 ± 0.84 b	0.6 ± 0.55 b
LP	0.8 ± 0.84 b	18 ± 0.71 a	17.4 ± 0.55 a	0.6 ± 0.55 b	0.8 ± 0.84 b

WT and transgenic seedlings were grown under SP or LP conditions for 6 days. Seedlings exhibiting root curling were counted. Values are the means ± SD (n=20). The experiment was repeated five times.
