# Supplementary material for: Overexpression of the protein phosphatase 2A regulatory subunit a gene ZmPP2AA1 improves low phosphate tolerance by remodeling the root system architecture of maize
Source: PLoS One. 2017 Apr 27;12(4):e0176538. doi: 10.1371/journal.pone.0176538 (PMC5407761; doi:10.1371/journal.pone.0176538)
Supplement: S1 Fig — Conserved residues between sequences are boxed in black or gray based on the degree of conservation. Arabidopsis: AtPP2AA1 (AT1G25490), AtPP2AA2 (AT3G25800) and AtPP2AA3 (AT1G13320); maize: ZmPP2AA1 (GRMZM2G164352), GRMZM2G102858, and GRMZM2G122135; rice: Osl_30535 and Os09g0249700; barley: MLOC_2967; Brachypodium: BRADI4G08720 and BRADI4G08790. (PDF) [file pone.0176538.s003.pdf]

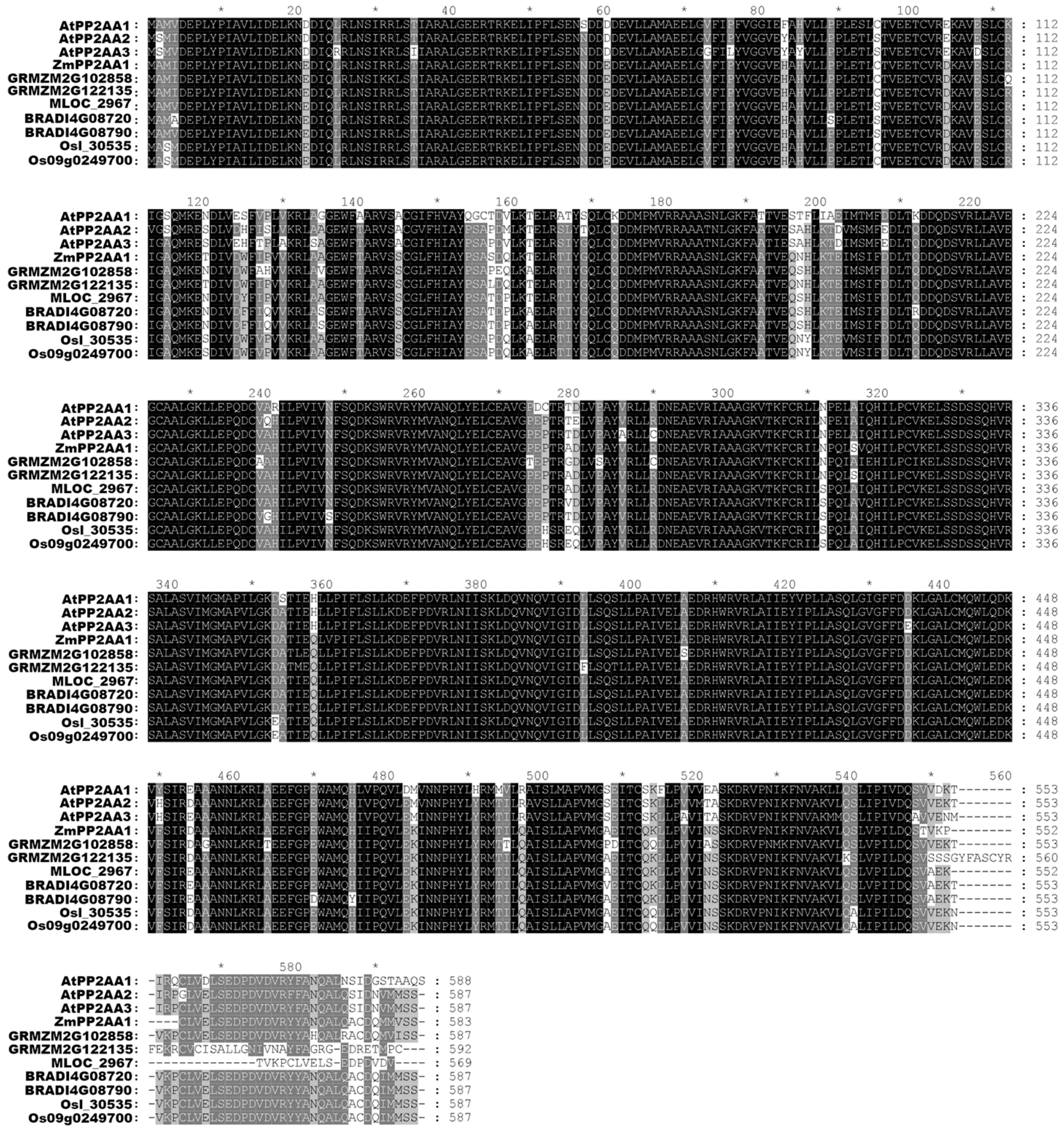

**S1 Figure. Alignment of the deduced amino acid sequence of PP2AAs between maize, *Arabidopsis*, rice, barley and *Brachypodium*.** Conserved residues between sequences are boxed in black or gray based on the degree of conservation. Arabidopsis: AtPP2AA1 (AT1G25490), AtPP2AA2 (AT3G25800) and AtPP2AA3 (AT1G13320); maize: ZmPP2AA1 (GRMZM2G164352), GRMZM2G102858, and GRMZM2G122135; rice: Osl_30535 and Os09g0249700; barley: MLOC_2967; *Brachypodium*: BRADI4G08720 and BRADI4G08790.
