# Supplementary material for: Overexpression of the protein phosphatase 2A regulatory subunit a gene ZmPP2AA1 improves low phosphate tolerance by remodeling the root system architecture of maize
Source: PLoS One. 2017 Apr 27;12(4):e0176538. doi: 10.1371/journal.pone.0176538 (PMC5407761; doi:10.1371/journal.pone.0176538)
Supplement: S2 Fig — (A) PCR analysis of the ZmPP2AA1 overexpressing T3 transgenic plants. M, DNA marker DL2,000; +, the PCR product of plasmid pCAMBIA3300-PUbi::ZmPP2AA1-Tnos-P35S::bar; -, the PCR product of H2O as a negative control template; WT, untransformed control Qi-319; OE-1, OE-4, OE-11, OE-15, OE-16, different ZmPP2AA1 overexpressing transgenic lines. (B) PCR analysis of ZmPP2AA1 RNAi T3 transgenic plants for the bar gene. M, DNA marker DL2,000; +, the PCR product of plasmid pCAMBIA3300-PUbi::zmpp2aa1-Tnos-P35S::bar as a positive control; WT, untransformed control Qi-319; RNAi-8, RNAi-10, RNAi-20, RNAi-22, RNAi-28, RNAi-32, different ZmPP2AA1 RNAi transgenic lines. (PDF) [file pone.0176538.s004.pdf]

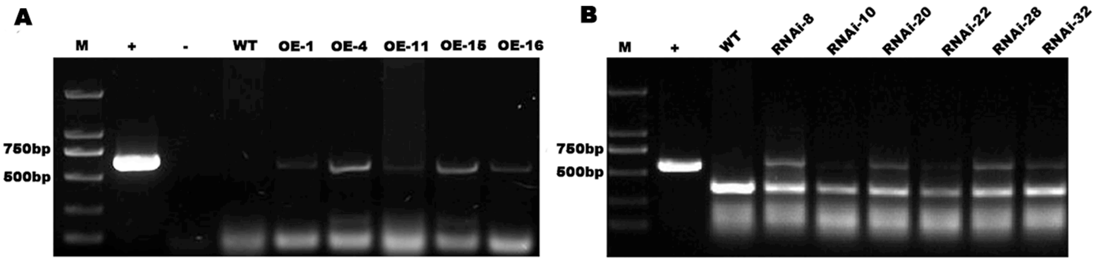

S2 Figure. PCR analysis of transgenic plants. (A) PCR analysis of the *ZmPP2AA1* overexpressing T_3 transgenic plants. M, DNA marker DL2,000; +, the PCR product of plasmid pCAM-BIA3300-PUbi::*ZmPP2AA1*-Tnos-P35S::*bar*; -, the PCR product of H_2O as a negative control template; WT, untransformed control Qi-319; OE-1, OE-4, OE-11, OE-15, OE-16, different *ZmPP2AA1* overexpressing transgenic lines. **(B)** PCR analysis of *ZmPP2AA1* RNAi T_3 transgenic plants for the *bar* gene. M, DNA marker DL2,000; +, the PCR product of plasmid pCAM-BIA3300-PUbi::*zmpp2aa1*-Tnos-P35S::*bar* as a positive control; WT, untransformed control Qi-319; RNAi-8, RNAi-10, RNAi-20, RNAi-22, RNAi-28, RNAi-32, different *ZmPP2AA1* RNAi transgenic lines.
