# Supplementary material for: Overexpression of the protein phosphatase 2A regulatory subunit a gene ZmPP2AA1 improves low phosphate tolerance by remodeling the root system architecture of maize
Source: PLoS One. 2017 Apr 27;12(4):e0176538. doi: 10.1371/journal.pone.0176538 (PMC5407761; doi:10.1371/journal.pone.0176538)
Supplement: S3 Fig — Bar = 5 cm. (PDF) [file pone.0176538.s005.pdf]

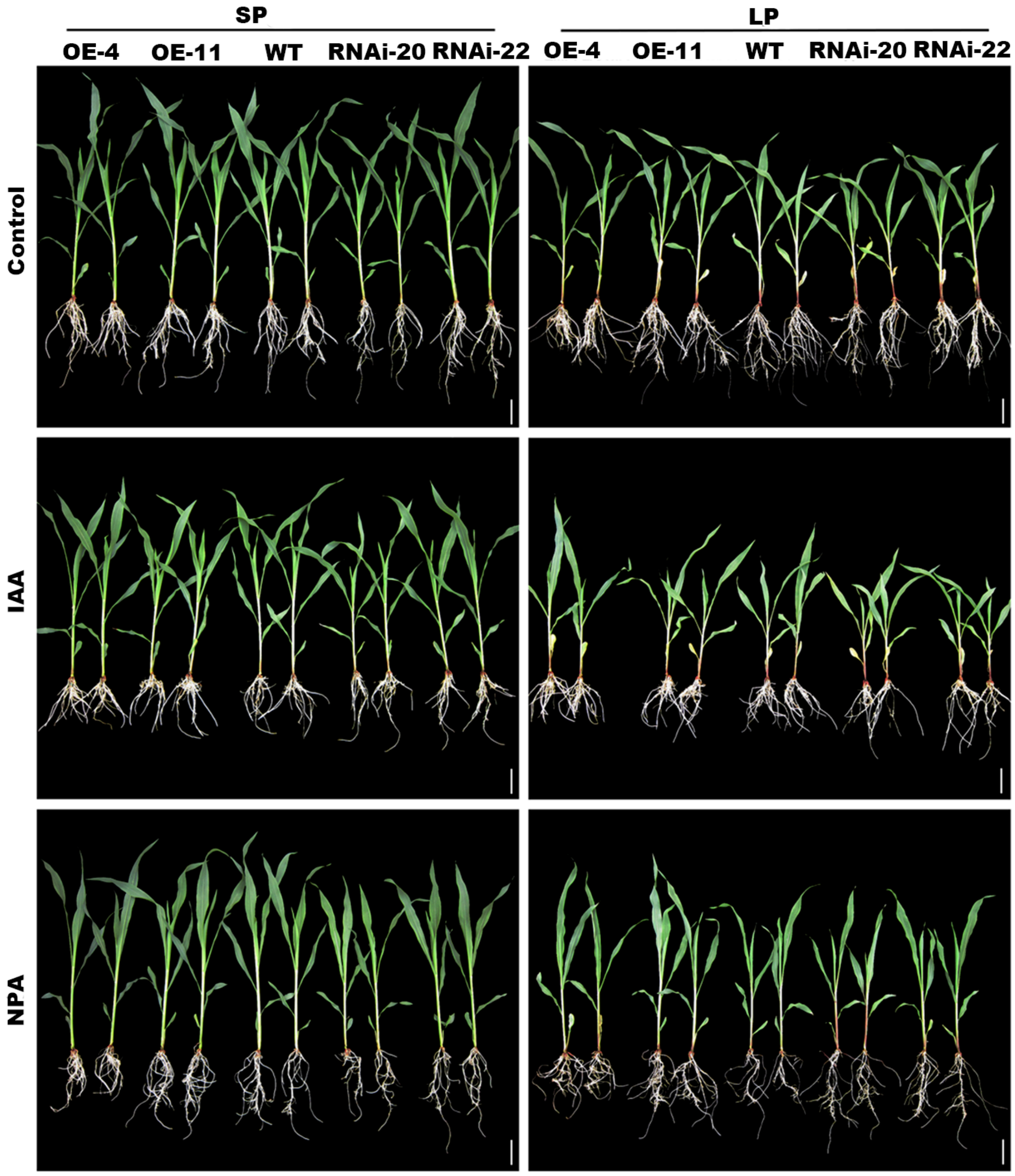

S3 Figure. Representative seedlings grown for 15d under SP (left) or LP (right) solution with or without IAA or NPA. Bar=5 cm.
